# Evaluation of the effect of the state of emergency for the first wave of COVID-19 in Japan

**DOI:** 10.1016/j.idm.2020.08.004

**Published:** 2020-08-17

**Authors:** Toshikazu Kuniya

**Affiliations:** Graduate School of System Informatics, Kobe University, 1-1 Rokkodai-cho, Nada-ku, Kobe, 657-8501, Japan

**Keywords:** COVID-19, SEIR epidemic model, Basic reproduction number, State of emergency

## Abstract

In this paper, we evaluate the effect of the state of emergency for the first wave of COVID-19 in Japan, 2020 from the viewpoint of mathematical modelling. In Japan, it was announced during the period of the state of emergency from April 7 to May 25, 2020 that the 80% reduction of the contact rate is needed to control the outbreak. By numerical simulation, we show that the reduction rate seems to have reached up to 86%. Moreover, we estimate the control reproduction number $R_{c}$ during the period of the state of emergency as $R_{c}=0.36$ (95%CI, 0.34–0.39), and show that the effective reproduction number $R_{e}$ after the lifting of the state of emergency could be greater than 1. This result suggests us that the second wave of COVID-19 in Japan could possibly occur if any effective intervention will not be taken again.

## 1 Introduction

The first case of novel coronavirus disease 2019 (COVID-19) was identified in Wuhan City, Hubei Province of China on December 31, 2019 (WHO, 2020, Situation report 1). As of June 30, 2020, the total number of globally reported cases and deaths of COVID-19 are 10,185,374 and 503,862, respectively (WHO, 2020, Situation Report 162).

**Fig. 1:**
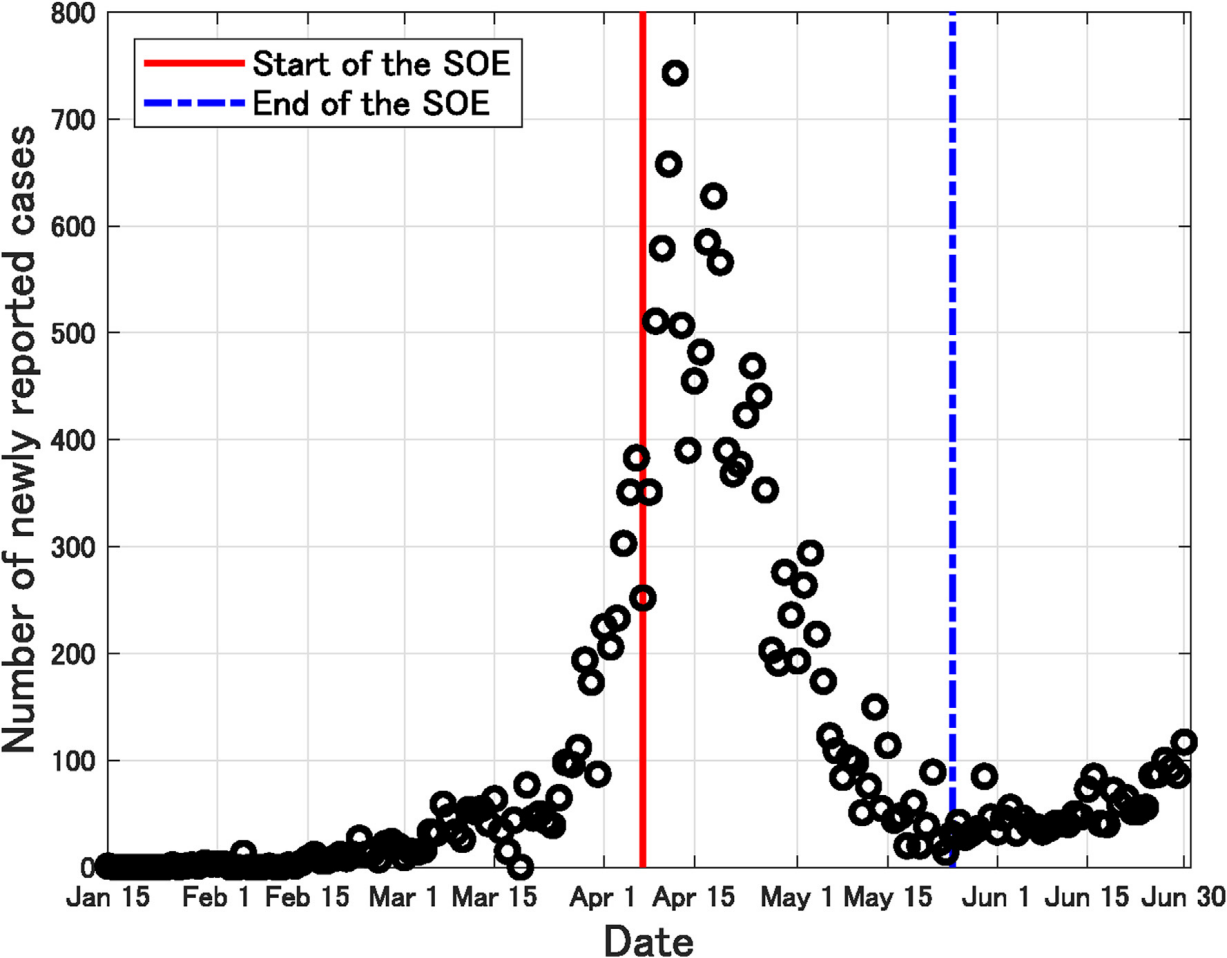
Daily number of newly reported cases of COVID-19 in Japan from January 15 to June 30, 2020. The start and end of the state of emergency (SOE) are on April 7 and May 25, 2020, respectively.

The first case of COVID-19 in Japan was identified on January 15, 2020 (WHO, 2020, Situation report 1). The increasement of the daily number of newly reported cases was observed in late February, and the early intervention such as school closure started from the beginning of March (The Japan Times, 2020). The serious exponential growth of the daily number of newly reported cases started from late March, and the Japanese government declared a state of emergency on April 7, 2020 (Kyodo News, 2020). After that, the daily number of newly reported cases tended to decrease, and the state of emergency was lifted on May 25, 2020 (Kyodo News, 2020). As of June 30, 2020, the daily number of newly reported cases in Japan is kept in a low level (see Fig. 1).

In this paper, we call the epidemic of COVID-19 in Japan until May 25, 2020 *the first wave of COVID-19 in* *Japan, 2020.*

Because the testing rate was low and the lockdown was insufficient (request-based) in Japan, it has been wondered why Japan succeeded in passing the first wave of COVID-19 (Foreign Policy, 2020). In Japan, it was announced in April after the declaration of the state of emergency that 80% reduction of the contact rate is needed to control the outbreak (Nikkei Asian Review, 2020). To the author’s knowledge, the majority of Japanese people seemed to keep the social distancing and the self-isolation following this announcement. In fact, it has been reported that the number of people who visited major tourist spots in Japan during the Japanese Golden week holidays (from late April to early May) in 2020 drastically decreased compared to that in 2019 (The Japan News, 2020). In areas around Ise Jingu Shrine, Mie Prefecture, it has been reported that more than 95% reduction was achieved (The Japan News, 2020). The purpose of this study is to evaluate the effect of the state of emergency for the first wave of COVID-19 in Japan from the viewpoint of mathematical modelling. In particular, our attention is on whether the 80% reduction of the contact rate was successfully achieved in Japan during the period of the state of emergency. For some prior studies on the effect of the control strategies for COVID-19 in Japan, see (Chen et al., 2020; Kobayashi et al., 2020; Kurita et al., 2020; Sugishita et al., 2020).

**Fig. 2:**
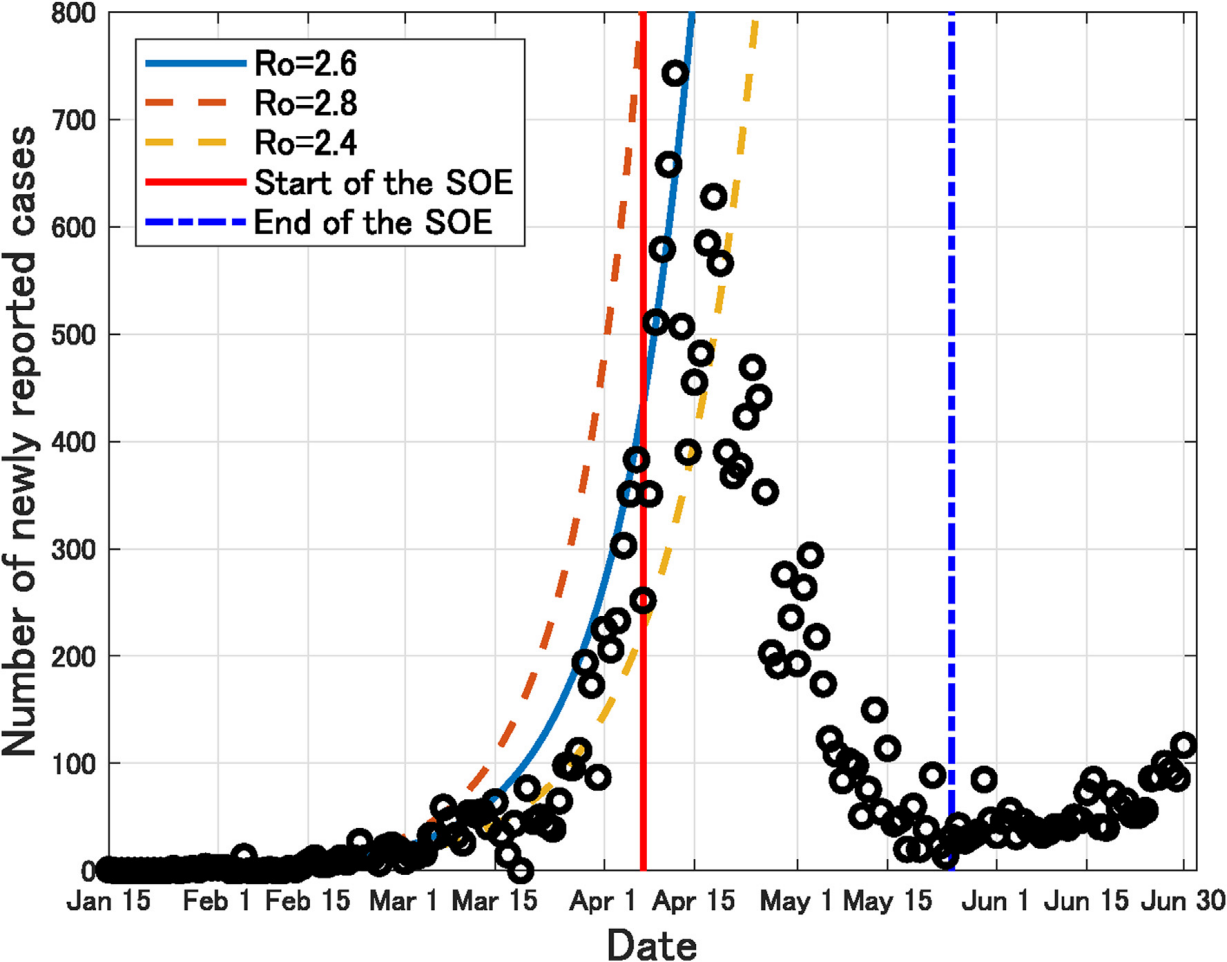
Comparison of the actual data of COVID-19 in Japan, 2020 and the predicted epidemic curve for $R_{0}=2.6$ (95%CI, 2.4–2.8), which was estimated in (Kuniya, 2020) using the early data (from January 15 to February 29, 2020).

In (Kuniya, 2020), the author estimated the epidemic parameters and predicted the epidemic peak for COVID-19 in Japan, 2020 by using the data in the early stage (from January 15 to February 29, 2020). The basic reproduction number $R_{0}$, which implies the expected number of secondary cases produced by a typical infected individual at the initial stage in a completely susceptible population (Diekmann et al., 1990), was estimated as 2.6 (95%CI, 2.4–2.8). The estimated epidemic curve in (Kuniya, 2020) seems to fit well to the actual data until about 2 weeks passed from the start of the state of emergency on April 7, 2020 (see Fig. 2).

From late April, the estimated epidemic curve has left the actual data. We can conjecture that if the state of emergency had not been declared on April 7, then the daily number of newly reported cases might have increased along with the estimated epidemic curve.

In this paper, we assume that the infection (contact) rate is successfully reduced during the period of the state of emergency (that is, from April 7 to May 25, 2020) by multiplying a constant k(0<k<1) to the infection rate. We manipulate the epidemic curve by changing *k*, and find the best $k=k^{\text{∗}}$ by which the epidemic curve is fitted well to the actual data. $\left(1-k^{\text{∗}}\right)\times 100$ (%) would then be the desired estimated value of the reduction rate of the contact rate during the period of the state of emergency for the first wave of COVID-19 in Japan, 2020.

## 2 Methods

**Fig. 3:**
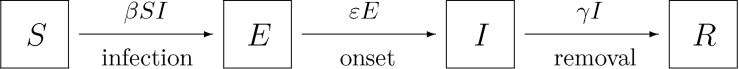
Transfer diagram for the SEIR model.

In prior studies, various compartmental models have been used to study COVID-19 (for instance, SIQR model (Crokidakis, 2020a, b), SIRX model (Maier & Brockmann, 2020) and SIRD model (Reis et al., 2020)). As the incubation period of COVID-19 is not negligible (Linton et al., 2020), there would be a merit for using an SEIR model, in which the latent class *E* is taken into consideration. In this paper, as in (Kuniya, 2020), we use the following SEIR model (see Fig. 3) with the detected infective population *Y*.

(1) $$S^{\prime }\left(t\right)=-\beta S\left(t\right)I\left(t\right),\;\;\;E^{\prime }\left(t\right)=\beta S\left(t\right)I\left(t\right)-\varepsilon E\left(t\right),\;\;\;I^{\prime }\left(t\right)=\varepsilon E\left(t\right)-\gamma I\left(t\right),\;\;\;R^{\prime }\left(t\right)=\gamma I\left(t\right),\;\;\;Y\left(t\right)=pI\left(t\right)N,$$

where *S*, *E*, *I* and *R* denote the susceptible, exposed, infective and removed populations, respectively. *β*, *ε*, *γ* and *p* denote the infection rate, the onset rate, the removal rate and the detection rate, respectively.

As stated below, each population implies the fraction to the total population. Hence, we can fit the daily data of newly reported cases by Y=pIN, where *N* denotes the total population in Japan. The baseline values of each parameter are as shown in Table 1.

**Table 1:** Baseline values of each parameter for model (1).

Parameter	Description	Value	Reference
*t*	Time	0-365 (days)	–
*S*	Susceptible population	0–1	–
*E*	Exposed population	0–1	–
*I*	Infective population	0–1	–
*R*	Removed population	0–1	–
*Y*	Detected infective population	pIN	–
*N*	Total population in Japan	$1.26\times 10^{8}$	SBJ (2020)
$R_{0}$	Basic reproduction number	2.6 (95%CI, 2.4–2.8)	Kuniya (2020)
*β*	Infection rate	0.26 (95%CI, 0.24–0.28)	Kuniya (2020)
1/ε	Average incubation period	5 (days)	Linton et al. (2020)
1/γ	Average infection period	10 (days)	Anderson et al. (2020)
*p*	Detection rate	0.25	Bommer and Vollmer (2020)

The initial condition is given as follows.

$$S\left(0\right)=1-I\left(0\right),\;I\left(0\right)=\frac{1}{pN},\;E\left(0\right)=R\left(0\right)=0.$$

This implies that one infective individual is confirmed at t=0 (that is, Y(0)=pI(0)N=1) and each population indicates the fraction to the total population as S(t)+E(t)+I(t)+R(t)=1 for all t≥0. The basic reproduction number $R_{0}$ is calculated as $R_{0}=\beta /\gamma$.

Let the unit time be 1 day and regard t=0 as January 15, 2020. Let $T_{1}=\left[0,83\right]$ be the time period before the state of emergency was declared on April 7 (t=83), and let $T_{2}=(83,131]$ be the time period during the state of emergency, which was lifted on May 25 (t=131). We assume that the epidemic process obeys the model (1) for $t\in T_{1}$, whereas it obyes the following alternative model for $t\in T_{2}$:

(2) $$S^{\prime }\left(t\right)=-k\beta S\left(t\right)I\left(t\right),\;\;\;E^{\prime }\left(t\right)=k\beta S\left(t\right)I\left(t\right)-\varepsilon E\left(t\right),\;\;\;I^{\prime }\left(t\right)=\varepsilon E\left(t\right)-\gamma I\left(t\right),\;\;\;R^{\prime }\left(t\right)=\gamma I\left(t\right),\;\;\;Y\left(t\right)=pI\left(t\right)N,$$

where 0<k<1. That is, the infection rate *β* is reduced to kβ during the period $T_{2}$ of the state of emergency. For each *k*, we define the following weighted least squares function as in (Capaldi et al., 2012, Section 3). Here, to specify the dependence on *k*, we write Y(t)=Y(t;k):

$$L\left(k\right):=\sum_{t\in T_{2}\cap N}\frac{{\left[Y\left(t;k\right)-Z\left(t\right)\right]}^{2}}{Y\left(t;k\right)},$$

where Z(t) denotes the actual number of newly reported cases at time *t*, which is collected from the situation reports in (WHO, 2020). We then find $k=k^{\text{∗}}$ that minimizes L(k).

## 3 Results

### 3.1 Estimation of the effect of the state of emergency

**Fig. 4:**
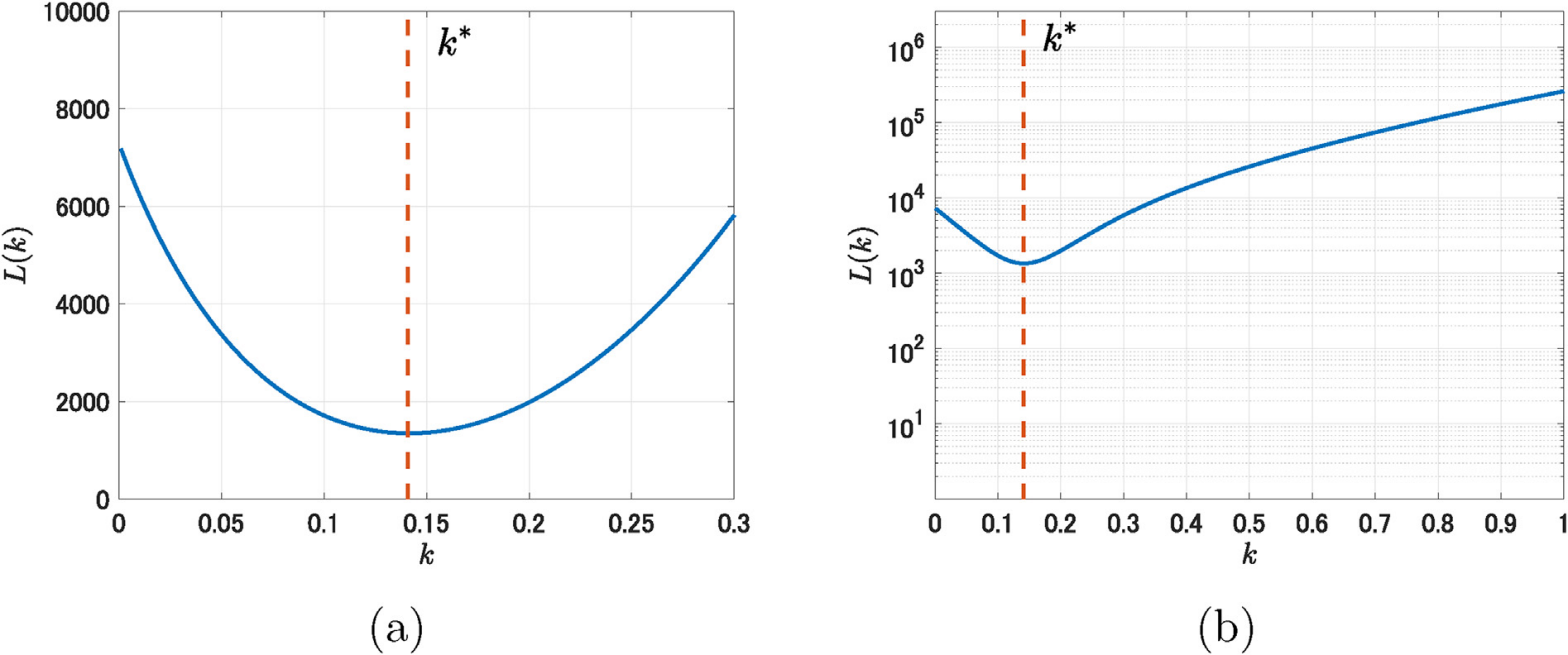
The weighted least square function L(k) versus 0<k<1.

**Fig. 5:**
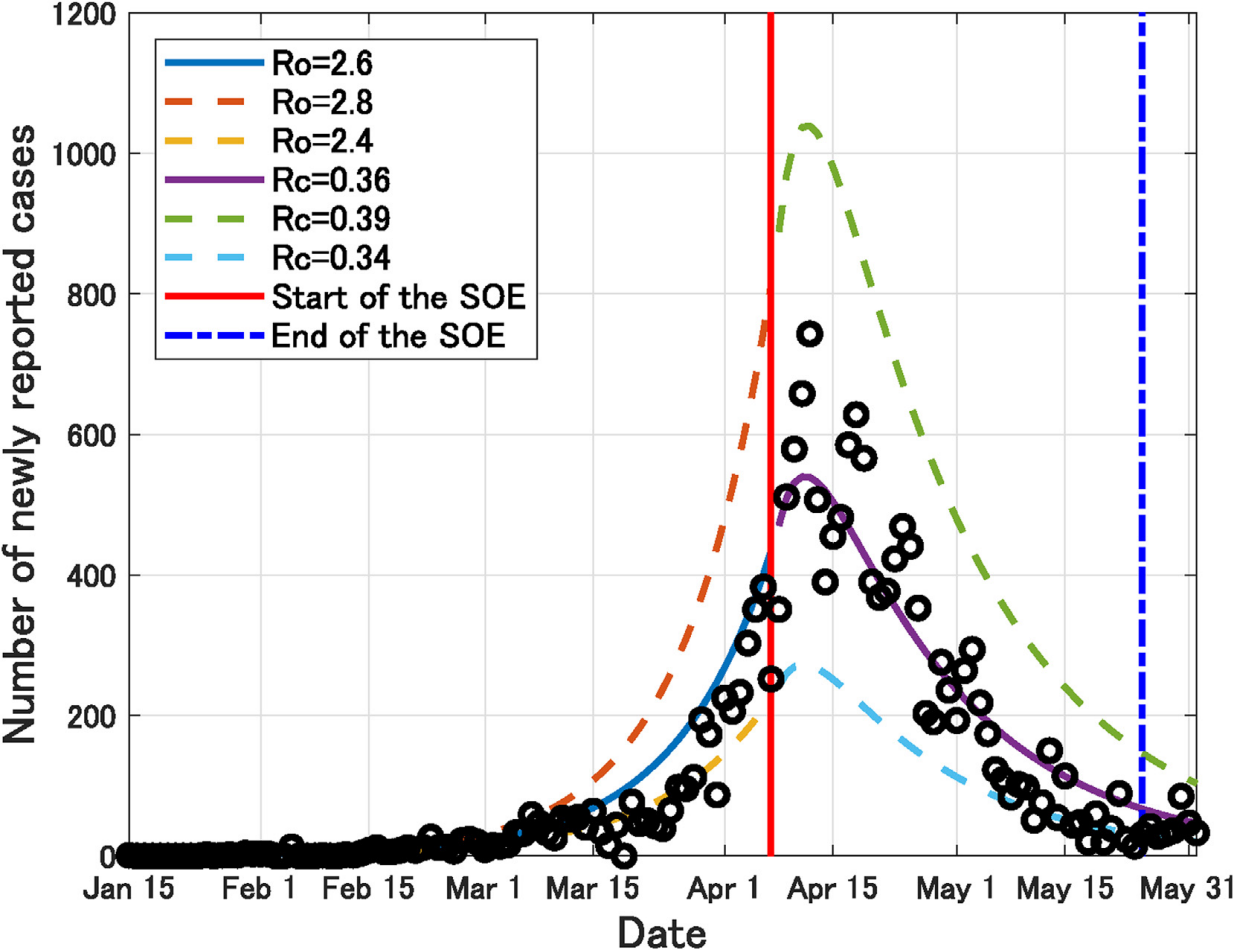
Comparison of the daily number of newly reported cases of COVID-19 in Japan, 2020 and the estimated epidemic curve with model (1) for $t\in T_{1}$ and model (2) for $t\in T_{2}$ ($k^{\text{∗}}=0.14$).

The weighted least square function L(k) is numerically calculated as in Fig. 4. From Fig. 4, we see that $k=k^{\text{∗}}=0.14$ minimizes L(k). The fitted epidemic curve for $k=k^{\text{∗}}=0.14$ is shown in Fig. 5. Here, $R_{c}$ denotes the control reproduction number (Inaba, 2017, Section 5.5.3), which is given by $R_{c}=k^{\text{∗}}R_{0}\approx 0.36$ (95%CI, 0.34–0.39). This result suggests us that the state of emergency in Japan for the first wave of COVID-19 resulted in $\left(1-k^{\text{∗}}\right)\times 100=86$% reduction of the contact rate.

### 3.2 Possibility of the second wave

**Fig. 6:**
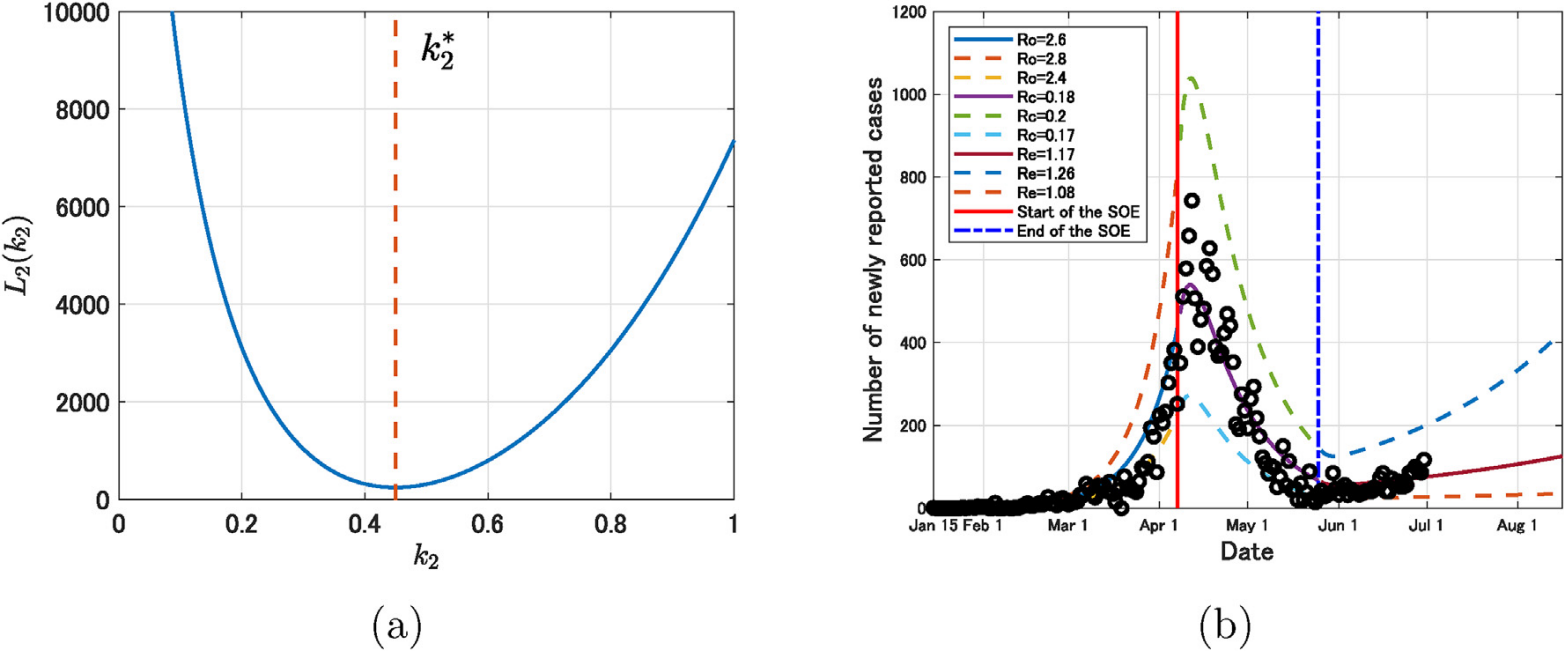
(a) The weighted least square function $L_{2}\left(k_{2}\right)$ versus $0<k_{2}<1$; (b) Comparison of the daily number of newly reported cases of COVID-19 in Japan, 2020 and the estimated epidemic curve with model (1) for $t\in T_{1}$, model (2) for $t\in T_{2}$ and model (3) for $t\in T_{3}\cup (167,365]$ ($k^{\text{∗}}=0.14$ and $k_{2}^{\text{∗}}=0.45$).

We next discuss the possibility of the second wave of COVID-19 in Japan. For some prior studies on the second wave of COVID-19, see, e.g., (Faranda & Alberti, 2020; Friston et al., 2020; Pires et al., 2020; Strzelecki, 2020). We use the data from May 25 (t=131) to June 30 (t=167) to estimate the epidemic curve after the lifting of the state of emergency. Let $T_{3}:=(131,167]$ and assume that the epidemic process obyes the following model for $t\in T_{3}\cup (167,365]$:

(3) $$S^{\prime }\left(t\right)=-k_{2}\beta S\left(t\right)I\left(t\right),\;\;\;E^{\prime }\left(t\right)=k_{2}\beta S\left(t\right)I\left(t\right)-\varepsilon E\left(t\right),\;\;\;I^{\prime }\left(t\right)=\varepsilon E\left(t\right)-\gamma I\left(t\right),\;\;\;R^{\prime }\left(t\right)=\gamma I\left(t\right),\;\;\;Y\left(t\right)=pI\left(t\right)N,$$

where $0<k_{2}<1$. As in Section 3.1, we define the weighted least square function

$$L_{2}\left(k_{2}\right):=\sum_{t\in T_{3}\cap N}\frac{{\left[Y\left(t;k_{2}\right)-Z\left(t\right)\right]}^{2}}{Y\left(t;k_{2}\right)},$$

and find $k_{2}=k_{2}^{\text{∗}}$ that minimizes $L_{2}\left(k_{2}\right)$. By numerical calculation as in Section 3.1, we obtain $k_{2}^{\text{∗}}=0.45$ (see Fig. 6 (a)). Now, we call $R_{e}:=k_{2}^{\text{∗}}R_{0}\approx 1.17$ (95%CI, 1.08–1.26) the effective reproduction number as of June 30, 2020 in Japan after the lifting of the state of emergency on May 25, 2020. As $R_{e}>1$ it seems to be possible that the second wave of COVID-19 in Japan will occur (see Fig. 6 (b)).

## 4 Discussion

In this paper, we have evaluated the effect of the state of emergency for the first wave of COVID-19 in Japan, 2020 by using the SEIR epidemic model (1)–(2). We have obtained $k^{\text{∗}}=0.14$, which implies that 86% reduction of the contact rate was achieved during the period of the state of emergency in Japan. On the other hand, we have obtained $k_{2}^{\text{∗}}=0.45$, which implies that the effective reproduction number $R_{e}$ as of June 30, 2020 after the lifting of the state of emergency on May 25, 2020 is greater than 1, and the second wave of COVID-19 in Japan could possibly occur. To avoid this worse scenario, some strong intervention might be required again.

Our simulation was based on the assumption that $R_{0}=2.6$ (95%CI, 2.4–2.8), which was estimated in (Kuniya, 2020). This assumption could be reasonable because the epidemic curve in Fig. 2, which was estimated by using the early data until February 29, 2020, seems to fit well to the data before the large intervention started on April 7, 2020. For the readers’ convenience, we refer to the estimated values of $R_{0}$ for COVID-19 in some prior studies (see Table 2).

**Table 2:** The estimated values of $R_{0}$ for COVID-19 in prior studies.

$R_{0}$	Country	Reference
5.25	Brazil	Crokidakis (2020a, b)
3.28 (average of estimations in 12 studies)	China	Liu et al. (2020)
4.50±1.44	European Union	Linka et al. (2020)
4.5	Global (across many nations)	Katul et al. (2020)
2.56	India	Rai et al. (2020)
2.7 (95%CI, 2.1–3.4)	Iran	Khosravi et al. (2020)
2.6 (95%CI, 2.3–2.9) or 3.3 (95%CI, 3.0–3.6)	Italy	Zhuang et al. (2020)
1.954 (95%CI, 1.851–2.025)	Japan	Chen et al. (2020)
1.49 (95%CI, 1.30–1.70)	Japan	Kobayashi et al. (2020)
2.86 (95%CI, 2.73–2.97)	Japan	Kurita et al. (2020)
2.50 (95%CI, 2.43–2.55)	Japan	Sugishita et al. (2020)
2.6 (95%CI, 2.3–2.9) or 3.2 (95%CI, 2.9–3.5)	Republic of Korea	Zhuang et al. (2020)
4.234 (95%CI, 3.764–4.7)	Russia	Nabi (2020)
4.02 (95%CI, 3.69–5.15)	USA	Gunzler (2020)

From Table 2, we can conjecture that $R_{0}$ for COVID-19 in Japan could be lower than the average in the world.

## 5 Conclusions

The conclusions in this paper are as follows.

• The 80% reduction of the contact rate in Japan seems to have been successfully achieved during the period of the state of emergency from April 7 to May 25, 2020. More precisely, the reduction rate seems to have reached up to 86%.
• The control reproduction number $R_{c}$ during the period of the state of emergency in Japan was estimated as $R_{c}=0.36$ (95%CI, 0.34–0.39).
• The effective reproduction number $R_{e}$ as of June 30, 2020 after the lifting of the state of emergency on May 25, 2020 seems to be greater than 1. This implies that the second wave of COVID-19 in Japan could possibly occur if any effective intervention will not be taken again.

The actual future pattern of COVID-19 might be unpredictable because it would be affected by many factors such as the social behavior and the number of PCR tests. However, our result suggests that the state of emergency might have been highly effective on the first wave of COVID-19 in Japan. If the second wave becomes realistic in Japan, then taking a strong intervention again without any hesitation could be important to avoid a catastrophic scenario.

## Declaration of competing interest

The author declares no conflict of interest.

## References

[bib1] Anderson R.M., Heesterbeek H., Kilnkenberg D. (2020). How will country-based mitigation measures influence the course of the COVID-19 epidemic?. The Lancet.

[bib2] Bommer C., Vollmer S. (2020). Average detection rate of SARS-CoV-2 infections is estimated around six percent. https://www.uni-goettingen.de/en/606540.html.

[bib3] Capaldi A., Behrend S., Berman B. (2012). Parameter estimation and uncertainty quantification for an epidemic model. Mathematical Biosciences and Engineering.

[bib4] Chen Z., Yang J., Dai B. (2020). Forecast possible risk for COVID-19 epidemic dissemination under current control strategies in Japan. International Journal of Environmental Research and Public Health.

[bib5] Crokidakis N. (2020). COVID-19 spreading in Rio de Janeiro, Brazil: Do the policies of social isolation really work?. Chaos, Solitons & Fractals.

[bib6] Crokidakis N. (2020). Modeling the early evolution of the COVID-19 in Brazil: Results from a susceptible-infectious-quarantined-recovered (SIQR) model. International Journal of Modern Physics C.

[bib7] Diekmann O., Heesterbeek J.A.P., Metz J.A.J. (1990). On the definition and the computation of the basic reproduction ratio *R*_0_ in models for infectious diseases in heterogeneous populations. J*ournal of Mathematical Biology*.

[bib8] Faranda D., Alberti T. (2020). Modelling the second wave of COVID-19 infections in France and Italy via a stochastic SEIR model. arXxiv.

[bib9] Friston K.J., Parr T., Zeidman P. (2020). Second waves, social distancing, and the spread of COVID-19 across America. Wellcome Open Research.

[bib10] Gunzler D.D., Sehgal A.R. (2020). Time-varying COVID-19 reproduction number in the United States. medRxiv.

[bib11] Inaba H. (2017). Age-structured population dynamics in demography and epidemiology.

[bib12] Katul G., Mrad A., Bonetti S. (2020). Global convergence of COVID-19 basic reproduction number and estimation from early-time SIR dynamics. medRxiv.

[bib13] Khosravi A., Chaman R., Rohani-Rasaf M. (2020). The basic reproduction number and prediction of the epidemic size of the novel coronavirus (COVID-19) in Shahroud, Iran. Epidemiology and Infection.

[bib14] Kobayashi G., Sugasawa S., Tamae H., Ozu T. (2020). Predicting intervention effect for COVID-19 in Japan: State space modeling approach. BioScience Trends.

[bib15] Kuniya T. (2020). Prediction of the epidemic peak of coronavirus disease in Japan, 2020. Journal of Clinical Medicine.

[bib16] Kurita J., Sugawara T., Ohkusa Y. (2020). Forecast of the COVID-19 outbreak and effects of self-restraint in going out in Tokyo, Japan. medRxiv.

[bib17] Kyodo News (2020). Abe declares coronavirus emergency over in Japan. https://english.kyodonews.net/news/2020/05/a1f00cf165ae-japan-poised-to-end-state-of-emergency-over-coronavirus-crisis.html.

[bib18] Linka K., Peirlinck M., Kuhl E. (2020). The reproduction number of COVID-19 and its correlation with public health interventions. medRxiv.

[bib19] Linton K.M., Kobayashi T., Yang Y. (2020). incubation period and other epidemiological characteristics of 2019 novel coronavirus infections with right truncation: A statistical analysis of publicly available case data. Journal of Clinical Medicine.

[bib20] Liu Y., Gayle G.A., Wilder-Smith A., Rocklöv J. (2020). The reproductive number of COVID-19 is higher compared to SARS coronavirus. International Society of Travel Medicine.

[bib21] Maier B.F., Brockmann D. (2020). Effective containment explains subexponential growth in recent confirmed COVID-19 cases in China. Science.

[bib22] Nabi K.N. (2020). Forecasting COVID-19 pandemic: A data-driven analysis. Chaos, Solitons & Fractals.

[bib23] Nikkei Asian Review (2020). Japan aims to cut social contact by 80%, here’s why. https://asia.nikkei.com/Spotlight/Coronavirus/Japan-aims-to-cut-social-contact-by-80-here-s-why.

[bib24] Pires M.A., Crokidakis N., Cajueiro D.O. (2020). What is the potential for a second peak in the evolution of SARS-CoV-2 in Brazil? Insights from a SIRASD model considering the informal economy.

[bib25] Foreign Policy (2020). Japan’s halfhearted coronavirus measures are working anyway. https://foreignpolicy.com/2020/05/14/japan-coronavirus-pandemic-lockdown-testing/.

[bib26] Rai B., Shkla A., Dwivedi L.K. (2020). COVID-19 in India: Predictions, reproduction number and public health preparedness. medRxiv.

[bib27] Reis R.F., Quintela B.M., Campos J.O. (2020). Characterization of the COVID-19 pandemic and the impact of uncertainties, mitigation strategies, and underreporting of cases in South Korea, Italy, and Brazil. Chaos, Solitons & Fractals.

[bib28] Statistics Bureau Japan (2020). Population estimates monthly report. http://www.stat.go.jp/english/data/jinsui/tsuki/index.html.

[bib29] Strzelecki A. (2020). The second worldwide wave of interest in coronavirus since the COVID-19 outbreaks in South Korea, Italy and Iran: A google trends study. Brain, Behavior, and Immunity.

[bib30] Sugishita Y., Kurita J., Sugawara T., Ohkusa Y. (2020). Preliminary evaluation of voluntary event cancellation as a countermeasure against the COVID-19 outbreak in Japan as of 11 March, 2020. medRxiv.

[bib31] The Japan News (2020). Golden Week holiday crowds down drastically from 2019 in Japan. https://the-japan-news.com/news/article/0006532389.

[bib32] The Japan Times (2020). Almost 99% of Japan’s public elementary schools shut as COVID-19 spreads. https://www.japantimes.co.jp/news/2020/03/05/national/99-japan-elementary-schools-close-doors-coronavirus/.

[bib33] WHO (2020). Coronavirus disease (COVID-2019) situation reports. https://www.who.int/emergencies/diseases/novel-coronavirus-2019/situation-reports.

[bib34] Zhuang Z., Zhao S., Lin Q. (2020). Preliminary estimates of the reproduction number of the coronavirus disease (COVID-19) outbreak in Republic of Korea and Italy by 5 March 2020. International Journal of Infectious Diseases.

